# Identification of TSG101 Functional Domains and p21 Loci Required for TSG101-Mediated p21 Gene Regulation

**DOI:** 10.1371/journal.pone.0079674

**Published:** 2013-11-11

**Authors:** Yu-Shiuan Lin, Yin-Ju Chen, Stanley N. Cohen, Tzu-Hao Cheng

**Affiliations:** 1 Institute of Biochemistry and Molecular Biology, National Yang-Ming University, Taipei, Taiwan; 2 Department of Genetics, Stanford University School of Medicine, Stanford, California, United States of America; Florida International University, United States of America

## Abstract

TSG101 (tumor susceptibility gene 101) is a multi-domain protein known to act in the cell nucleus, cytoplasm, and periplasmic membrane. Remarkably, TSG101, whose location within cells varies with the stage of the cell cycle, affects biological events as diverse as cell growth and proliferation, gene expression, cytokinesis, and endosomal trafficking. The functions of TSG101 additionally are recruited for viral and microvesicle budding and for intracellular survival of invading bacteria. Here we report that the TSG101 protein also interacts with and down-regulates the promoter of the p21*^CIP1/WAF1^*tumor suppressor gene, and identify a p21 locus and TSG101 domains that mediate this interaction. TSG101 deficiency in Saos-2 human osteosarcoma cells was accompanied by an increased abundance of p21 mRNA and protein and the retardation of cell proliferation. A *cis*-acting element in the p21 promoter that interacts with TSG101 and is required for promoter repression was located using chromatin immunoprecipitation (ChIP) analysis and p21-driven luciferase reporter gene expression, respectively. Additional analysis of TSG101 deletion mutants lacking specific domains established the role of the central TSG101 domains in binding to the p21 promoter and demonstrated the additional essentiality of the TSG101 C-terminal steadiness box (SB) in the repression of p21 promoter activity. Neither binding of TSG101 to the p21 promoter nor repression of this promoter required the TSG101 N-terminal UEV domain, which mediates the ubiquitin-recognition functions of TSG101 and its actions as a member of ESCRT endocytic trafficking complexes, indicating that regulation of the p21 promoter by TSG101 is independent of its role in such trafficking.

## Introduction

*TSG101* (Tumor susceptibility gene 101) was identified initially in mouse cells in a genetic screen for loci that modulate neoplastic transformation of NIH3T3 fibroblasts and tumor formation in nude mice [1]. Down-regulation of *TSG101* expression and adventitious overexpression of the gene were both found to produce a neoplastic phenotype [1], and subsequent studies have provided further evidence that regulation of cell proliferation by TSG101 is bidirectional and multifaceted [2]–[4].

More than 15 years of study has revealed that TSG101 affects multiple cellular functions beyond growth and proliferation; these include gene expression, cytokinesis, and endosomal trafficking [5]–[9]. In mice, null mutation of *TSG101* is lethal during early stages of embryogenesis [2], [5]. The protein is present in both the cell nucleus and cytoplasm, and its distribution within cells varies according to stage of the cell cycle [4]. The functions of TSG101 additionally are recruited for viral and microvesicle budding and for intracellular survival of invading bacteria [10], [11].

The 46 KDa TSG101 protein contains a domain that is characteristic of ubiquitin conjugases but lacks a site necessary for it to function as an agent of ubiquitination; additional distinct domains in the protein are the proline-rich (PR), coiled-coil, and steadiness box (SB) regions [12]. The functions of the individual domains of the TSG101 protein, and especially those of the ubiquitin E2 variant (UEV) domain, have been studied extensively. As a component of the ESCRT-1 complex, TSG101 regulates endosomal trafficking of ubiquitinated proteins by interacting with PTAP or PSAP motifs of these proteins through its UEV domain [8], [13]. Human immunodeficiency virus and other retroviruses that encode proteins containing PTAP or PSAP motifs can hijack TSG101-dependent ESCRT-1 endosomal protein-sorting complexes to deliver virions to the plasma membrane [14], [15].

TSG101 also functions as a transcriptional regulator to suppress hormone-receptor-mediated transactivation of targeted genes, which requires interaction of nuclear hormone receptors with the centrally-located PR and coiled-coil domains of TSG101 [16], [17]. Protein-protein interactions involving these TSG101 domains may facilitate localization of TSG101 to the specific gene loci that it controls [16]. TSG101-containing protein complexes also have been implicated in transcriptional repression of chromatin loci affected by DNA methylation or histone acetylation [18].

An elevated cellular abundance of TSG101 protein normally is prevented post-translationally, at least in part through autoregulation by the C-terminal SB domain [19]. Adventitious production of TSG101 triggers polyubiquitylation of the SB by TAL (TSG101-associated ligase), which promotes TSG101 degradation [20]. However, increased abundance of TSG101 protein has been observed in cancer cells [6], [21], and the additional ability of antisense RNA or siRNA directed against *TSG101* to alter cellular functions [8] implies that autoregulation of TSG101 protein stability by the SB does not prevent the effects of perturbations that affect negatively *TSG101* expression at the pre-translational level.

At least some of the effects of TSG101 on cell proliferation have been attributed its actions on the cyclin-dependent kinase inhibitor p21 *^CIP1/WAF1^*, which negatively regulates cell cycling [22], [23]. TSG101 deficiency can increase expression of p21 mRNA and protein [2], [6], [24]. However, the mechanism underlying such increase has been unclear, as the TSG101 protein lacks a sequence having features characteristic of a canonical DNA binding motif. Here we report that domains located near the center of the TSG101 protein enable TSG101 to interact with to a 200 bp region of the p21 promoter, that such binding represses p21 promoter activity, and that repression of p21 promoter activity is independent of the UEV domain that is central to TSG101 function in endocytic trafficking.

## Materials and Methods

### Plasmid construction

A DNA fragment spanning the human p21 promoter from -3227 to the transcription start site +1 was PCR-amplified and cloned into pGL2 (Promega) to create the (-3227/+1) p21-Luc reporter. (-2325/+1) p21-Luc was similarly generated and described previously [25]. 5′ serial deletion constructs, including (-2125/+1) p21-Luc, (-1928/+1) p21-Luc, (-1727/+1) p21-Luc, (-1518/+1) p21-Luc, (-1326/+1) p21-Luc and (-1125/+1) p21-Luc, were derived from (-2325/+1) p21-Luc. (-2325/+1Δd) p21-Luc lacking identified TSG101 binding site was created by deletion of sequences between -1864 and -1568 of (-2325/+1) p21-Luc. pcDNA-SRα/HA vector was kindly provided by Dr. Y.-H. W. Lee. pcDNA-SRα/HA-TSG101 was constructed by subcloning a *TSG101* open reading frame from pLLEXP-Tsg*-Flag [8] that contains silent mutations and therefore is resistant to TSG101-specific shRNA. Constructs expressing Myc-tagged full-length TSG101 and truncated forms have been described [12].

### Cell culture and transfection

Saos-2 and U2OS cells were obtained from the American Type Culture Collection. HCT116 and the isogenic cell line HCT116 p21^-/-^ [26], [27] were kindly provided by Dr. Jim Ford. Cells were grown in Dulbecco's modified Eagle's medium (Saos-2, TSG101 knockdown cell lines, and U2OS) or McCoy's 5A (HCT116 and HCT116 p21^-/-^) supplemented with 10% fetal bovine serum. Transfections were carried out using Lipofectamine 2000 (Invitrogen) as per the manufacturer.

Stable TSG101 knockdown cell lines were established in Saos-2 cells constitutively expressing TSG101 shRNA. A construct with shRNA targeting the 413-433 coding sequence of the gene and a pGFP-IRES-Puro plasmid were introduced into Saos-2 cells by co-transformation and colonies exhibiting both puromycin resistance and GFP green fluorescence were isolated, cloned, and assayed for TSG101 expression. Two clones showed decreased TSG101 abundance to ∼50% of the level observed in parental cells.

### Western blot analysis and Antibodies

Western blot analysis was performed as described [28], except that RIPA buffer contained 1% SDS for cell lysis. Antibodies against TSG101 (C-2, Santa Cruz), p21 (CP74, Sigma), β-tubulin (D-10, Santa Cruz), β-actin (AC-15, Abcam), Nucleophosmin/B23 (FC-61991, Zymed Laboratories), PARP (MA3-950, Thermo Scientific), and activated Caspase-3 (AB3623, Chemicon) were purchased, as was monoclonal anti-Myc antibody (LTK Biolaboratories), which was used for chromatin immunoprecipitation.

### Real-time quantitative RT-PCR

As described [28], an equivalent amount of total RNA from each sample was converted into cDNA and PCR-amplified to determine relative transcript levels. Quantitative real-time PCR was performed using a StepOnePlus Real Time PCR system (Applied Biosystem) and analyzed using the Quantitative-Comparative C_T_ (ΔΔC_T_) program. PCR primer set (p21 qPCR-5′ and p21 qPCR-3′) was used for p21, and (β-actin qPCR-5′ and β-actin qPCR-3′) was used for β-actin, which served as an internal control. Oligonucleotide sequences are listed in Table S1.

### Reporter assay for p21 promoter activity

Saos-2-2 cells were co-transfected with p21 reporter constructs and empty pcDNA-SRα/HA vector or the TSG101 expression construct pcDNA-SRα/HA-TSG101. A plasmid expressing β-galactosidase (β-gal) was included as an internal control. 24 hr post transfection, cells were lysed using the freeze-thawing method in 100 µl of lysis buffer (0.25 M Tris-HCl [pH7.8], 100 mM potassium phosphate buffer [pH7.8], 3 mM MgCl_2_, 1 mM DTT, 0.1% NP-40 and protease inhibitor cocktail). Cell extracts (60 µl) were then mixed with 250 µl luciferase assay buffer (43.2 mM glycylglycine [pH 7.8], 22 mM MgSO_4_, 2.4 mM EDTA, 7.4 mM ATP, 1 mM DTT, and 0.4 mg of bovine serum albumin), and 100 µl of 0.5 mM luciferin (Promega). Luciferase activity was measured using an AutoLumat LB953 luminometer (Berthold) and normalized to β-galactosidase activity. Statistical analysis of data was performed by two-tailed unpaired Student's *t* test. A *P* value of <0.05 was considered significant.

In order to examine the role of chromatin modification, Saos-2-2 cells were co-transfected with a reporter construct (-1727/+1) p21-Luc and empty vector or a TSG101 expression construct, and then treated with 0.5 µM TSA or 100 µM RG108. TSA and RG108 stock solutions were dissolved in ethanol.

### Chromatin immunoprecipitation (ChIP)

ChIP assays were performed as described [29], [30]. Briefly, cells were fixed with 1% formaldehyde, washed with ice-cold PBS, and resuspended in cell lysis buffer (5 mM PIPES, 85 mM KCl, and 0.5% NP-40 plus a protease inhibitor cocktail). After centrifugation, the nuclear pellet was collected and resuspended in nuclear lysis buffer (50 mM Tris-HCl [pH8.0], 10 mM EDTA, and 0.2% SDS plus a protease inhibitor cocktail), followed by sonication using a microtip ultrasonicator. Nuclear lysates were then mixed with an equal volume of IP buffer (16.7 mM Tris-HCl [pH8.0], 167 mM NaCl, 0.01% SDS, 1.1% Triton X-100 and 1.2 mM EDTA), pre-cleared with protein A-Sepharose beads, and incubated with monoclonal antibody specific for TSG101 or the Myc-epitope at 4°C overnight. Immunocomplexes were recovered by adding protein A-Sepharose beads and incubating samples at 4°C for 2 hr. Beads were washed twice with the following buffers: 1^st^, IP buffer; 2^nd^, IP buffer containing 500 mM NaCl; 3^rd^, 10 mM Tris-HCl, 0.25 M LiCl, 0.5% NP-40, 1 mM EDTA and 0.5% sodium deoxycholate; and 4^th^, 1x TE buffer. Precipitated DNA fragments were eluted from beads after RNase treatment, followed by proteinase K digestion in the presence of SDS (0.25%) and de-crosslinking by incubation at 65°C. Following phenol/chloroform extraction, DNA was resuspended in 50 µl TE buffer. 5 µl of the DNA solution was PCR-amplified using p21 promoter-specific primer sets (Table S1).

### Subcellular fractionation

Subcellular fractionation was performed as described [31] with minor modification. Cells were suspended in buffer A (10 mM HEPES [pH7.9], 10 mM KCl, 1.5 mM MgCl_2_, 1.5 mM DTT, and 5% glycerol) at a ratio of 100 mg cells per 800 µl buffer. The sample was incubated on ice for 5 min to lyse cells after addition of Triton X-100 (C_f_ = 0.1%). Following centrifugation, the nuclear pellet was collected and the supernatant saved as the cytoplasmic fraction. The nuclear pellet was further rinsed in ice-cold PBS, re-suspended in RIPA buffer supplemented with 1 mM Na_3_VO_4_, 1 mM DTT, 1 mM PMSF and a protease inhibitor cocktail (Sigma), and sonicated. After centrifugation (14,000 *g* for 20 min), the supernatant was saved as the nuclear fraction.

### siRNA knockdown

HCT116 and HCT116 p21^-/-^ cells were transfected with siRNA using LipofectAMINE 2000. To inhibit TSG101expression, 60 nM of TSG101-specific siRNA (5′-CCUCCAGUCUUCUCUCGUCTT-3′ and 5′-GACGAGAGAAGACUGGAGGTT-3′) synthesized by DHARMACON was used. Transfection of an equivalent amount of annealed double-stranded oligonucleotides (5′-UUCUCCGAACGUGUCACGUTT-3′ and 5′-ACGUGACACGUUCGGAGAATT-3′), which do not target any gene, served as a negative control.

### Measurement of cell proliferation

Following transfection with TSG101-specific or control siRNA, HCT116 or HCT116 p21^-/-^cells were seeded at a density of 5×10^4^ in triplicate in 6-well plates. Cells were collected, stained with trypan blue, and counted by a hemocytometer every 24 hr for 3 days. Saos-2 cells and the TSG101-knockdown line Saos-2-2 were transfected with either pcDNA-SRα/HA vector or pcDNA-SRα/HA-TSG101. 36 hours later, cells were seeded at a density of 8×10^4^. Cell counting was performed as described above for 4 days. Statistical significance of the results was analyzed by Student's *t* test.

## Results

### p21 gene transcription is up-regulated in TSG101 deficient cells

**Figure 1:**
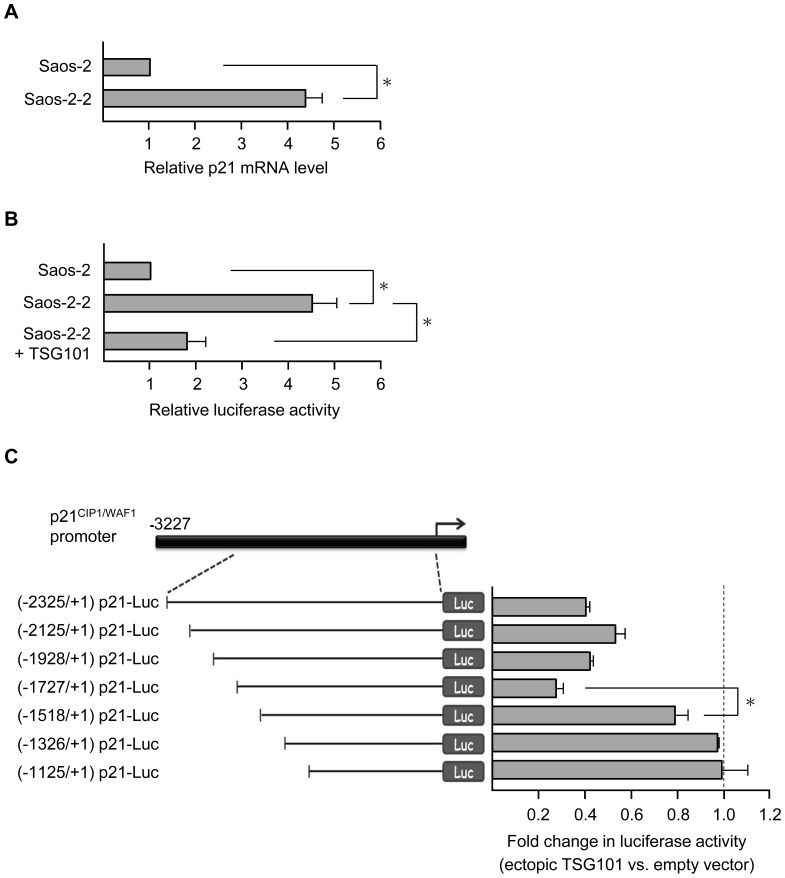
Stable TSG101 knockdown cell lines show increased p21*^CIP1/WAF1^* expression. (A) p21 mRNA levels in Saos-2 and Saos-2-2 cells were assessed by real-time quantitative RT-PCR. Transcripts were normalized to β-actin, and the p21 mRNA level in Saos-2 cells was set to 1. Saos-2-2 stable line shows 4.3±0.5 in relative p21 mRNA level. (B) p21 promoter activity was examined by luciferase reporter assay. Cells as indicated were transfected with the (-3227/+1) p21-Luc construct, in which the firefly luciferase gene is driven by the p21 promoter. Luciferase activities were measured and normalized to β-gal, and the activity in parental Saos-2 cells was set as 1. Relative luciferase activities of Saos-2-2 (4.5±0.55) or of Saos-2-2 cells expressing ectopic TSG101 (1.8±0.4) are shown. (C) Left, schematic diagram of reporter constructs with a series of p21 promoter deletions. The transcription start site is indicated by the rightward arrow in the top panel and numbered +1. Right, effect of TSG101 on promoter activity of p21 deletion constructs. Saos-2-2 cells were transfected with individual reporter plasmid together with an empty vector or a TSG101 expression construct pcDNA-SRα/HA-TSG101. The ratio of luciferase activity in cells with or without ectopic TSG101 expression is shown. Data presented are means ± s.d. of three independent experiments (*P<0.05, Student's *t* test).

To investigate cellular events associated with TSG101 deficiency, we established a stable line of Saos-2 cells (here designated as Saos-2-2) in which shRNA directed against *TSG101* mRNA reduced the abundance of TSG101 protein by approximately 50% as determined by Western blotting (data not shown). During microarray analysis of the effects of such reduction on global gene expression (Table S2), we observed that p21 mRNA abundance was several fold higher in Saos-2-2 than in the isogenic parental cell line. Real time RT-PCR analysis (Fig. 1A) showed a similar increase, and the ability of shRNA-induced TSG101 deficiency to elevate production of a luciferase reporter gene driven by the p21 promoter in Saos-2-2 cells established that transcription initiated at the p21 promoter activity is up-regulated in cells made deficient in TSG101 (Fig. 1B and Fig. S1). Luciferase elevation was partially reversed when pcDNA-SRα/HA-TSG101, which expresses a *TSG101* open reading frame containing translationally-silent nucleotide substitutions that make the adventitiously expressed ORF insensitive to inhibition by the shRNA sequence used to inhibit endogenous TSG101 expression [8] was introduced in the shRNA treated cells (Fig. 1B), confirming that the effects of the shRNA had resulted specifically from perturbation of TSG101 expression.

To identify the p21 promoter region required for TSG101-mediated regulation, a series of deletions in the p21 promoter was constructed, and luciferase expression from these constructs was measured in Saos-2-2 cells that overexpressed TSG101 from pcDNA-SRα/HA-TSG101. As shown in figure 1C, TSG101 overexpression inhibited promoter activity of (-2325/+1) p21-Luc, (-2125/+1) p21-Luc, (-1928/+1) p21-Luc, and (-1727/+1) p21-Luc but only minimally affected production of luciferase from (-1518/+1) p21-Luc. We conclude that a sequence responsive to TSG101-mediated regulation is located in the region between -1727 and -1518 upstream of the p21 transcription start site.

**Figure 2:**
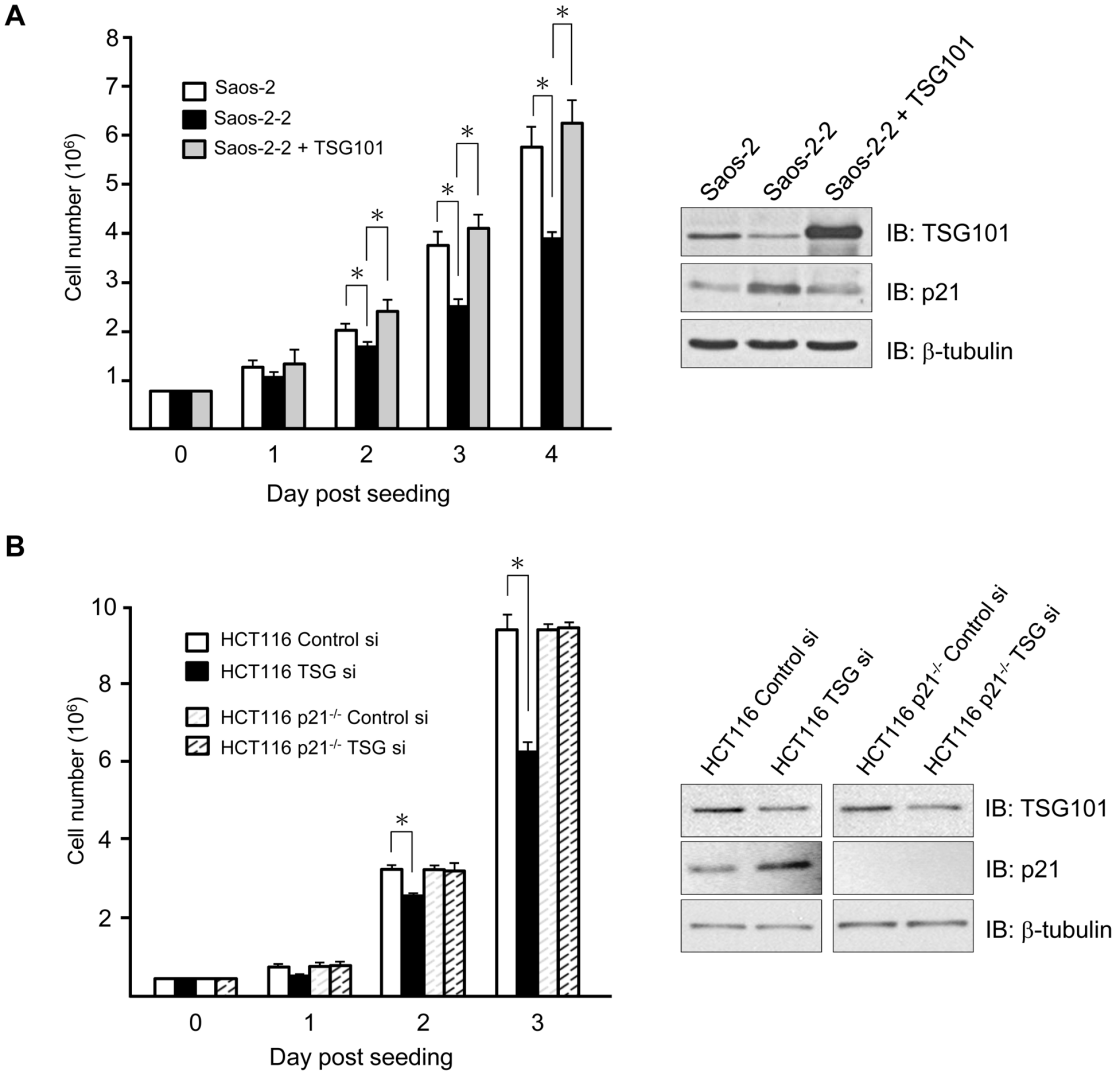
TSG101 deficiency causes cell growth defect via p21. (A) Left, proliferation of Saos-2, Saos-2-2, and Saos-2-2 cells expressing ectopic TSG101 was measured by counting viable cells for 4 consecutive days. Right, TSG101 and p21 protein levels were assessed by Western blot analysis in indicated cells. β-tubulin serves as a loading control. (B) HCT116 and isogenic HCT116 p21^-/-^ cells were subjected to TSG101-specific siRNA (TSG101 si) knockdown and changes in TSG101 and p21 protein levels relative to samples treated with control siRNA (Control Si) were analyzed by Western blot (Right). Cell growth was monitored for 3 days (Left). Results were the average of three independent experiments done in triplicate (*P<0.05, Student's *t* test).

Fibroblasts isolated from *TSG101* ^-/-^ mouse embryos and tumor cells treated with siRNA directed against *TSG101* have been reported to show reduction of the rate of cell growth [6], [32]. Similarly, we observed a correlation between TSG101 protein levels and cell proliferation in Saos-2 cell derivatives (Fig. 2A). The cell growth retardation effects associated with TSG101 knockdown were shown to be specifically dependent on the presence of p21 in experiments that employed HCT116 cells and an isogenic cell line in which the p21 gene had been deleted (HCT116 p21^-/-^ cells). A population of HCT116 cells transiently transfected with siRNA directed against *TSG101* showed a reduced rate of cell growth relative to control HCT116 cells, concurrent with an increase in p21 expression as assayed by Western blot analysis. In contrast, HCT116 p21^-/-^ cells lacking the p21 gene showed no effects of TSG101 knockdown on cell proliferation (Fig. 2B). Consistent with these findings, TSG101 up-regulation has been reported to be associated with faster cell proliferation and poor prognosis in ovarian malignancy, and an inverse correlation between TSG101 and p21 protein levels has been observed in such tumors [6].

### Identification of a TSG101 binding locus in the p21 promoter

**Figure 3:**
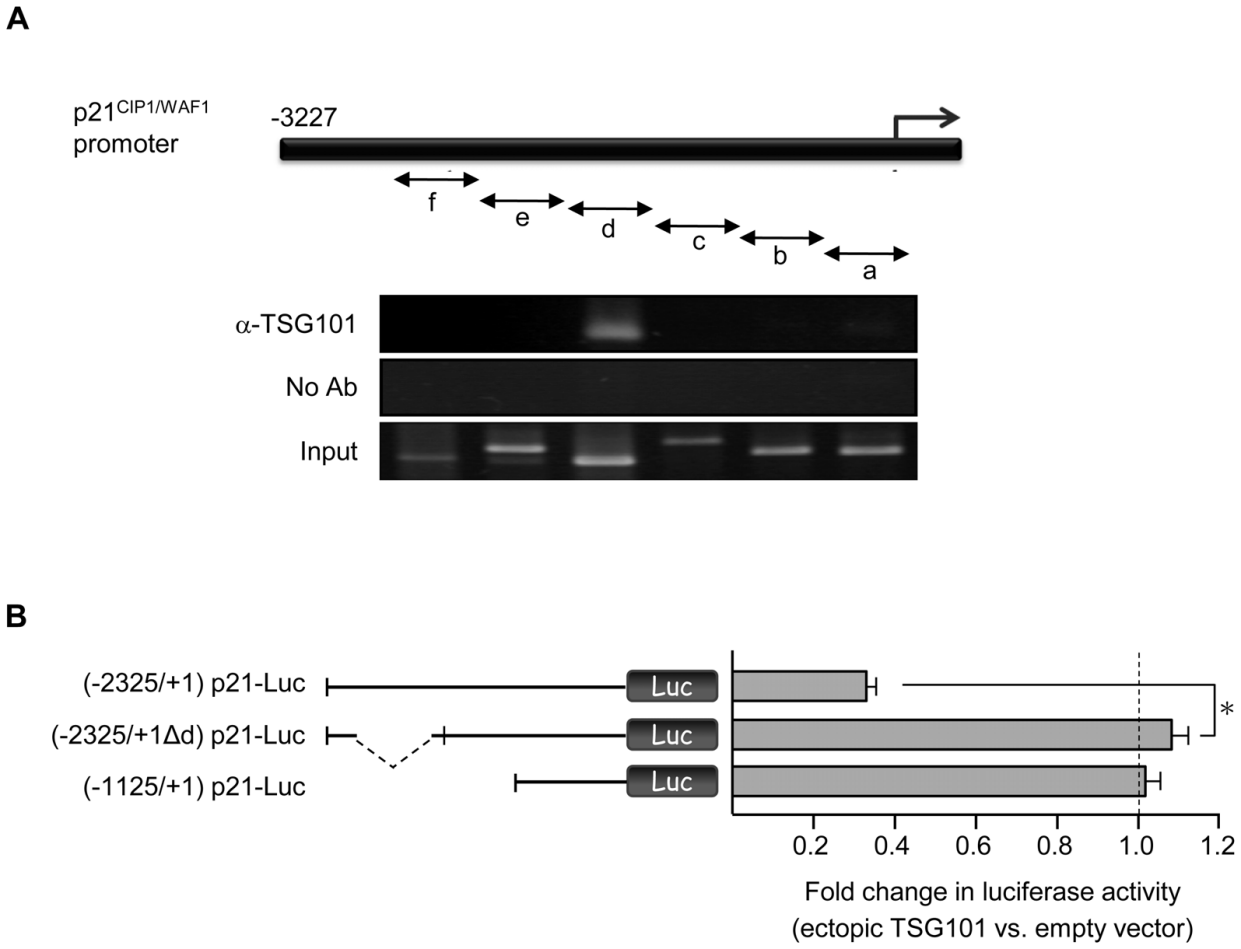
TSG101 binds to and negatively regulates the p21 promoter. (A) TSG101 occupancy of the p21 promoter was determined by chromatin immunoprecipitation in Saos-2 cells. Precipitated DNA fragments were examined using primer sets a, b, c, d, e, and f. Regions amplified by each primer set are indicated in the upper panel. Primer set “d” detects a region containing 1567∼2026 bp upstream of the transcription start site. (B) The repressive effect of TSG101 was examined using construct (-2325/+1Δd) p21-Luc, in which the TSG101 binding site is deleted, as shown in (A). The (-2325/+1) p21-Luc and (-1125/+1) p21-Luc constructs served as positive and negative controls, respectively. Values presented are means ± s.d. for three separate experiments (*P<0.05, Student's *t* test).

Chromatin immunoprecipitation (ChIP) assays showed that sequences of the p21 promoter region interact directly with the TSG101 protein and identified the TSG101-binding sequence in Saos-2 as the region amplified by primer set “d” (Fig 3A). TSG101 binding to this locus was also observed in HCT116 cells (data not shown). The TSG101-binding region identified by ChIP notably overlapped with the promoter region shown by deletion analysis (i.e., -1727 to -1518) (Fig. 1C) to be required for regulation of p21 promoter activity by ectopic overexpression of TSG101. Consistent with the conclusion that this region contains a sequence that interacts—either directly or indirectly—with TSG101 in cells and is regulated by such interaction, repression of p21 promoter-driven luciferase by ectopically-expressed TSG101 was abolished by deletion of the segment containing the p21 sequence identified as a TSG101 binding locus (Fig. 3B).

The possibility that TSG101 repression of the p21 promoter is affected by DNA methylation or histone acetylation, both of which can modulate the functions of a TSG101-co-repressor DMAP1 in a cell cycle dependent manner was tested by evaluating TSG101 mediated repression of the p21 promoter in the presence of the DNA methyltransferase inhibitor RG108 [33], [34] or the histone deacetylase inhibitor Trichostatin A (TSA) [35]. We found that p21 promoter activity was increased by TSA treatment as reported by others [36], and that this enhancement was suppressed by TSG101 expression. However, neither of the inhibitors affected the actions of TSG101 on the p21 promoter (Fig. S2).

### TSG101 domains are differentially required in the action of p21 promoter binding and repression

**Figure 4:**
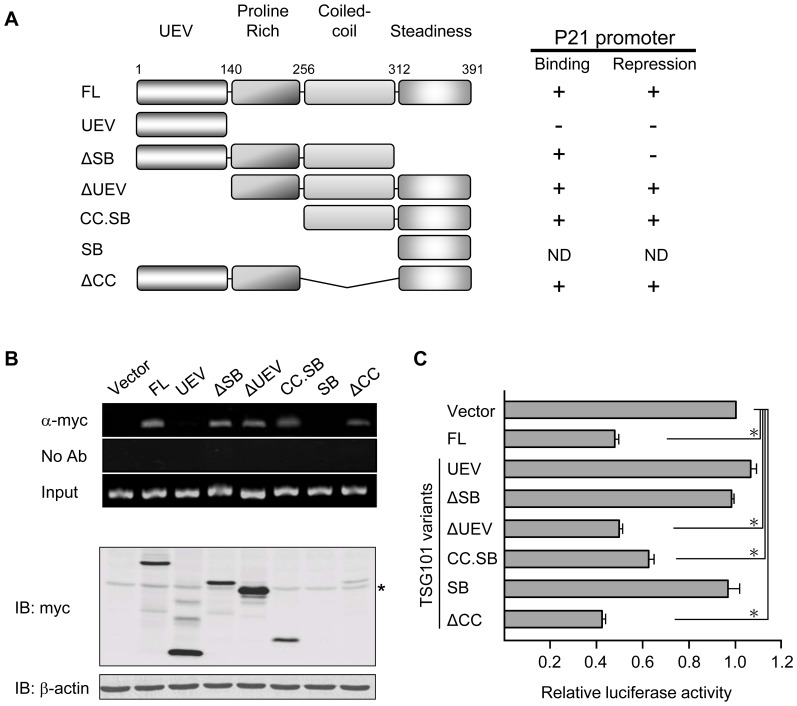
Specific TSG101 domains are required for p21 promoter binding and repression. (A) Left, TSG101 contains 391 amino acids with the four indicated domains. Constructs expressing myc-tagged full-length or truncated forms of TSG101 are illustrated. Right, p21 promoter binding and repression by these TSG101 variants are summarized. ND, not determined. (B) Binding of full-length TSG101 and its truncated forms to the p21 promoter was examined by chromatin immunoprecipitation. DNA fragments, which co-precipitated with anti-myc antibody, were PCR-amplified using the primer set “d”, as shown in Figure 3A. Expression of TSG101 variants is shown by the Western blot in the bottom panel. Asterisk indicates the position of non-specific bands. (C) p21 promoter activity was assessed in Saos-2-2 cells expressing different forms of TSG101. Empty vector sample was included as control, and its promoter activity was set as 1. Data presented are the average of five independent experiments (*P<0.05, Student's *t* test).

Variant *TSG01* transcripts lacking segments present in full-length *TSG101* mRNA have been reported to accumulate in multiple types of human cancer [37]–[39]. To identify the specific TSG101 region that interacts with and regulates the p21 promoter and to establish a framework for identifying possible effects of aberrantly spliced *TSG101* transcripts on p21-regulated cell proliferation, we constructed a collection of truncated variants of TSG101 (Fig. 4A, left panel). ChIP assays using these variants showed that the UEV domain alone (UEV) did not interact with the p21 promoter and that TSG101 protein lacking such domain (ΔUEV) retained the ability to bind. These results argue strongly that regulation of p21 promoter activity by TSG101 does not result from TSG101 interaction with PTAP or PSAP containing proteins, as TSG101 effects on endocytic trafficking do.

Extension of the ΔUEV deletion through the proline-rich domain (CC.SB) did not affect binding (Fig. 4B, top panel), indicating that this domain is not necessary for interaction of TSG101 with the p21 promoter. The CC and SB domains also are not required for interaction of TSG101 with the p21 promoter, as shown by the continued ability of the ΔCC and ΔSB mutants to bind (Fig 4B, top panel). However, failure of a construct containing only the UEV domain to bind indicates that at the presence of either the PR or CC domain enables binding. Collectively these findings indicate that TSG101 contains more than one locus that allows it to interact with the p21 promoter. A TSG101 variant containing only the SB domain was very poorly expressed (Fig. 4B, bottom panel), precluding meaningful analysis of its binding ability.

Testing of TSG101 variants for their ability to repress p21 promoter activity, as determined by the p21-luciferase reporter gene assay described above showed that expression of certain variant TSG101 proteins that did directly interact with the p21 promoter nevertheless decreased p21 promoter activity (Fig. 4C and 4A). For ΔUEV, CC.SB, and ΔCC variants, the decrease by the truncated and full-length proteins was comparable (Fig. 4C). However, TSG101 protein lacking the SB domain (ΔSB) did not affect p21 promoter activity (Fig. 4C) despite its ability to bind to the promoter (Fig. 4B).

### Identification of TSG101 domains that affect its cellular location

**Figure 5:**
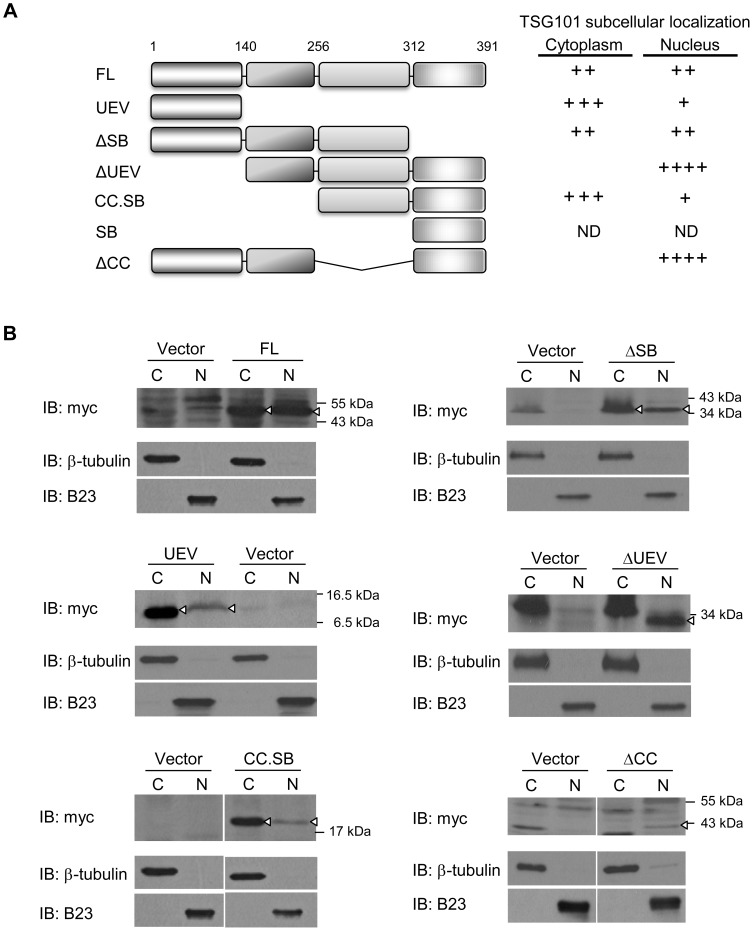
Subcellular localization of TSG101 variants. (A) The presence of TSG101 variants in the cytoplasm or nucleus is summarized. ND, not determined. (B) Nuclear (N) and cytoplasmic (C) fractions of U2OS cells expressing indicated constructs were examined by Western blot analysis. In each pair of samples, empty vector (Vector) was included as a control. Arrowheads indicate positions of TSG101 protein. B23 and β-tubulin serve as markers of nuclear and cytoplasmic fractions, respectively.

The distribution of TSG101 within cells has been shown to be cell cycle dependent, occurring in the nucleus and Golgi during interphase and in mitotic spindles, centrosomes, and in the midbody bridge between cells that is evident during the later stage of cytokinesis [4], [7]. The presence of TSG101 in the cell nucleus should be prerequisite for its binding to and/or repression of the p21 promoter and we wished to elucidate the roles of the various TSG101 domains in determining the sub-cellular location of TSG101 in cytoplasmic and nuclear preparations that had been obtained by fractionation of U2OS cell lysates. As expected from earlier work, full-length TSG101 (FL) was represented approximately equally in the nucleus and cytoplasm of an actively growing cell population (Fig. 5B). A similar protein distribution was observed for a TSG101 variant lacking the SB domain (ΔSB) (Fig. 5B). However, a variant TSG101 protein that includes only the UEV domain (UEV) was preferentially present in the cytoplasmic fraction, and conversely TSG101 lacking either this domain (ΔUEV) or the coiled-coil domain (ΔCC) accumulated in the nucleus (Fig. 5B). Extension of the ΔUEV deletion through the proline-rich domain (CC.SB) dramatically shifted the predominant cellular location of TSG101 from nucleus to cytoplasm (Fig 5B and 5A). These results indicate that multiple sites within the TSG101 protein have a role in determining its intracellular distribution. TSG101 was detected in the nucleus for all variants that showed the ability to bind to the p21 promoter.

## Discussion

The results reported here demonstrate that the cell cycle regulator and tumor suppressor gene p21 is negatively regulated by interaction of the TSG101 protein with the p21 promoter, leading to consequent suppression of p21-mediated cell growth retardation by adventitiously expressed TSG101. Our findings further indicate that binding of TSG101 to specific sequences in the p21 promoter is necessary but not sufficient for transcriptional repression of p21 expression.

Our results implicate TSG101 sequences other than the coiled-coil domain of TSG101, which contains a leucine zipper motif characteristic of transcriptional repressors [1], [16] in regulation of p21 promoter activity; at least two distinct sites located in the central region of TSG101 can interact with the p21 promoter and repress its function, implying that the TSG101/p21 promoter interaction may occur via multiple mechanisms or intermediaries. Interestingly, the central region of TSG101 previously was shown to interact with the AF-1 region of nuclear hormone receptors to repress transcription of targeted genes [40]. The UEV domain of TSG101, which is essential to TSG101 inclusion in endosomal trafficking complexes is not required for interaction of TSG101 with the p21 promoter or for repression of p21 promoter activity, making it unlikely that such complexes participate in TSG101 repression of p21 gene expression.

Multiple transcription activator/repressor proteins can modulate p21 gene expression; sites in the p21 promoter that interact with these proteins have been extensively characterized [41], [42], and the mechanisms of repression of p21 promoter activity by such proteins have been well studied [22]. Commonly, negative regulators (for example, proto-oncogene FBI-1 and c-Myc) that prevent p21 transcription either compete with positive regulators for promoter binding or interact with positive regulators to interfere with their ability to bind to targeted sites in DNA [22]. The region of the p21 promoter that our data shown is required for control of this promoter by TSG101 does not overlap with the p21-promoter-binding sites of any of the known transcriptional up-regulators of p21. Whether other proteins interact with TSG101 to assist with TSG101 binding to the p21 promoter or conversely, whether the TSG101 central domains can assist the binding of other proteins that regulate p21 promoter activity has not been determined. In any case, analysis by the BLAST program of NCBI did not reveal any additional DNA sequence in the human genome that resembles the region of the p21 promoter necessary for repression by TSG101. Moreover, binding sites for known human transcription factors were not identified within this TSG101-responsive DNA segment using the databases of TFSEARCH (http://www.cbrc.jp/research/db/TFSEARCH.html) and PROMO (http://alggen.lsi.upc.es/cgi-bin/promo_v3/promo/promoinit.cgi?dirDB=TF_8.3).

Our microarray data indicate that a subset of genes having distinct cellular functions related to p21 show altered gene expression as a consequence of TSG101 down-regulation. Among these, the p21 and cyclin-dependent kinase 6 (CDK6) are direct regulators of cell cycle progression and G1/S transition [43], [44]. Using HCT116 and the isogenic cell line HCT116 p21^-/-^, we have validated that at least the p21 gene is required for the defect of cell growth by TSG101 deficiency. In the stable line Saos-2-2, cell apoptosis does not substantially increase compared to its parental cells (Fig. S3); therefore, the relative slow proliferation of this cell is likely resulting from increased p21 expression, leading to a delay in cell cycle progression.

While multiple TSG101-deficient clones were obtained during our isolation of the Saos-2-2 stable line, none of these clones showed a reduction in TSG101 greater than 60%, suggesting that a baseline level of TSG101 is needed for cell growth and/or survival. This finding is consistent with earlier evidence that total elimination of TSG101 function is a lethal event [2], [9], [24]. The approach we used to isolate a Saos-2-2 stable line showing decreased TSG101 function was also applied unsuccessfully to another osteosarcoma cell line U2OS, which unlike Saos-2 synthesizes p53 protein [45]. It has been shown that TSG101 down-regulation can increase the amount and activity of p53 via MDM2 instability and also results in p53-dependent up-regulation of the p21 gene [2], [12]. We suggest that in p53-profieient cells the previously reported effects of TSG101 on p53, and consequently on p21 expression, may supplement the p21-dependent effects on cell proliferation reported here, and may thus account for the sensitivity of U2OS or other p53-proficient cells to experimental reduction of the cellular TSG101 protein level.

Aberrant splicing and production of *TSG101* transcript variations have been detected in multiple types of human cancer, including carcinomas of the liver, lung, breast, cervix, and ovary [46]-[50] as well as in soft tissue sarcomas [51], and leukemias [52]. It has been suggested that such aberrant transcripts are experimental artifacts of PCR analysis [53]; however, it also has been reported that induction of the tumor suppressor gene p53 prevents the accumulation of variant transcripts [54]. While differential effects of TSG101 isoforms on tumor development and progression have not been established, our results suggest that tumor associated isoforms that do not contain the central or SB domain of TSG101 would lack the ability of the full-length protein to repress the p21 promoter.

Our results demonstrate an inverse correlation between TSG101 and p21 protein levels. Using HCT116 p21^+/+^ and isogenic HCT116 p21^-/-^ cells, we’ve shown that proliferation defects associated with TSG101-deficiency are mediated at least in part by the modulation of p21 gene expression. Our findings thus indicate that TSG101 regulation of p21 is an important factor in the cellular function of TSG101, as has been suggested also by results showing that altered TSG101 expression in ovarian cancer may affect disease prognosis by modulating p21 expression [6].

## Supporting Information

Figure S1 **Luciferase reporter activity in p21 promoter-less construct is negligible and such low activity is not modulated by cellular TSG101 levels.** pGL2-basic, a reporter plasmid lacking the p21 promoter sequence, was transfected into Saos-2 and Saos-2-2 cells for luciferase reporter assay. pGL2-p21 (-3227/+1), in which the firefly luciferase gene is driven by the p21 promoter, was included as a positive control. Following β-gal normalization, the reporter activity of each sample is illustrated as Relative Luminescence Unit (RLU). The data presented are the average of three independent experiments (*P<0.05, Student's *t* test).
(TIF)

Figure S2 **p21 repression by TSG101 is maintained in the presence of Trichostatin A or RG108.** (A) p21 promoter activity in Saos-2-2 cells with or without ectopic TSG101 expression was assessed in the presence or absence of the histone deacetylase inhibitor Trichostatin A (TSA). p21 promoter activity of the mock sample transfected with the empty vector was set as 1. Relative promoter activity of the other samples is shown. (B) Relative p21 promoter activity was examined as described in (A), except the DNA methyltransferase inhibitor RG108 was used. The data presented are the average of three independent experiments (*P<0.05, Student's *t* test).
(TIF)

Figure S3 **Saos-2-2 does not show a substantial increase of cell apoptosis compared to its parental cells.** (A) Saos-2 and Saos-2-2 were analyzed for cell apoptosis by TUNEL assay. Saos-2 treated with Adriamycin (ADR, 1 µM), a DNA-damaging reagent, also were included as a positive control. Scale bars, 100 µm. (B) TSG101, Caspase-3, and Poly ADP-ribose polymerase (PARP) were examined in indicated cells by Western blot analyses. Activated Caspase-3 (17 KDa) and the cleavage form of PARP (85 KDa) both serve as index for the activation of apoptotic signal pathways.
(TIF)

Table S1 **Oligonucleotide primers used in Chromatin Immunoprecipitation (ChIP) and quantitative RT-PCR (qPCR).**
(PDF)

Table S2 **List of genes that are statistically significant up-regulated or down-regulated in Saos-2-2 compared with its parental cells.** Microarray data were deposited in GEO with an accession number GSE50808 (http://www.ncbi.nlm.nih.gov/geo/query/acc.cgi?acc=GSE50808), and analyzed by a software package “Limma” at http://bioconductor.org/packages/2.12/bioc/html/limma. html. Genes up-regulated or down-regulated more than 4-fold (with a *p* value less than 0.01) are shown.
(PDF)
