# Supplementary figures and images for: Identification of TSG101 Functional Domains and p21 Loci Required for TSG101-Mediated p21 Gene Regulation
Source: PLoS One. 2013 Nov 11;8(11):e79674. doi: 10.1371/journal.pone.0079674 (PMC3823576; doi:10.1371/journal.pone.0079674)

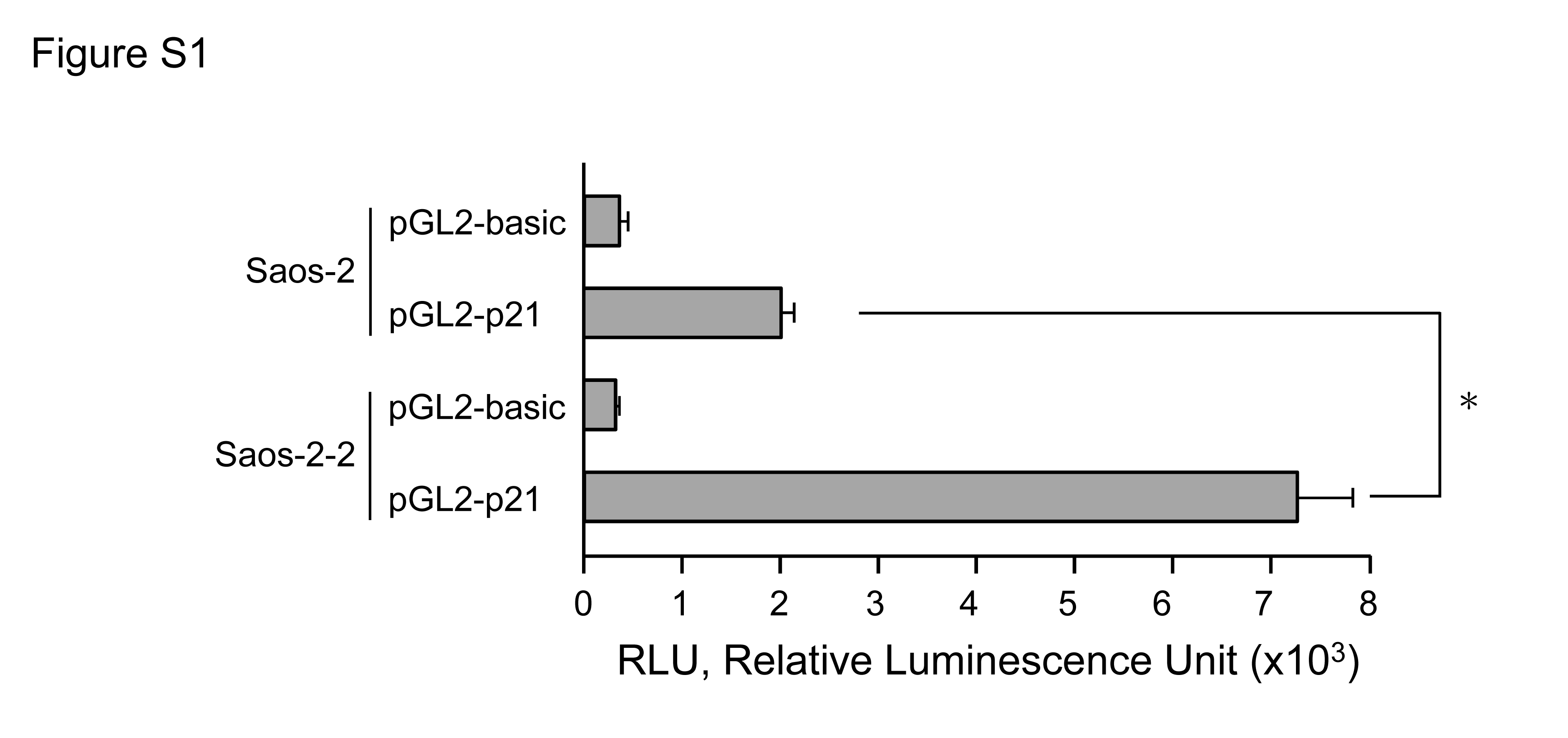

Supplement: Figure S1 — Luciferase reporter activity in p21 promoter-less construct is negligible and such low activity is not modulated by cellular TSG101 levels. pGL2-basic, a reporter plasmid lacking the p21 promoter sequence, was transfected into Saos-2 and Saos-2-2 cells for luciferase reporter assay. pGL2-p21 (-3227/+1), in which the firefly luciferase gene is driven by the p21 promoter, was included as a positive control. Following β-gal normalization, the reporter activity of each sample is illustrated as Relative Luminescence Unit (RLU). The data presented are the average of three independent experiments (*P<0.05, Student's t test). (TIF) [file pone.0079674.s001.tif]

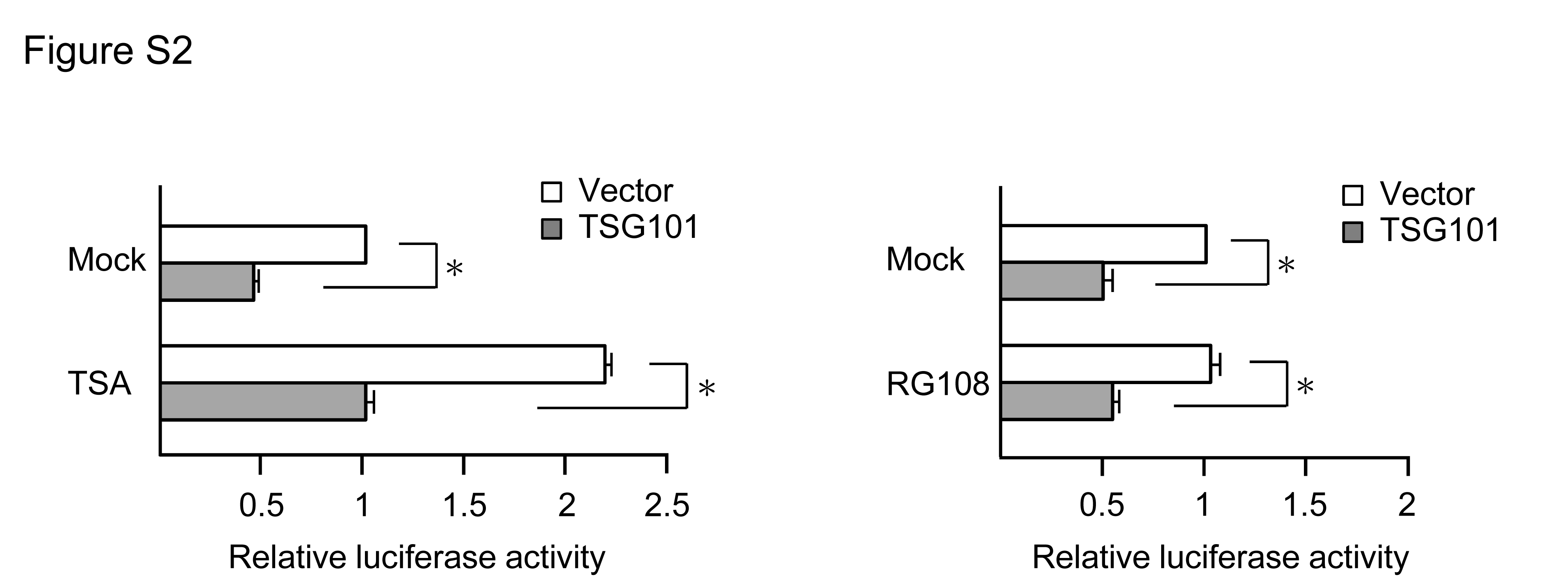

Supplement: Figure S2 — p21 repression by TSG101 is maintained in the presence of Trichostatin A or RG108. (A) p21 promoter activity in Saos-2-2 cells with or without ectopic TSG101 expression was assessed in the presence or absence of the histone deacetylase inhibitor Trichostatin A (TSA). p21 promoter activity of the mock sample transfected with the empty vector was set as 1. Relative promoter activity of the other samples is shown. (B) Relative p21 promoter activity was examined as described in (A), except the DNA methyltransferase inhibitor RG108 was used. The data presented are the average of three independent experiments (*P<0.05, Student's t test). (TIF) [file pone.0079674.s002.tif]

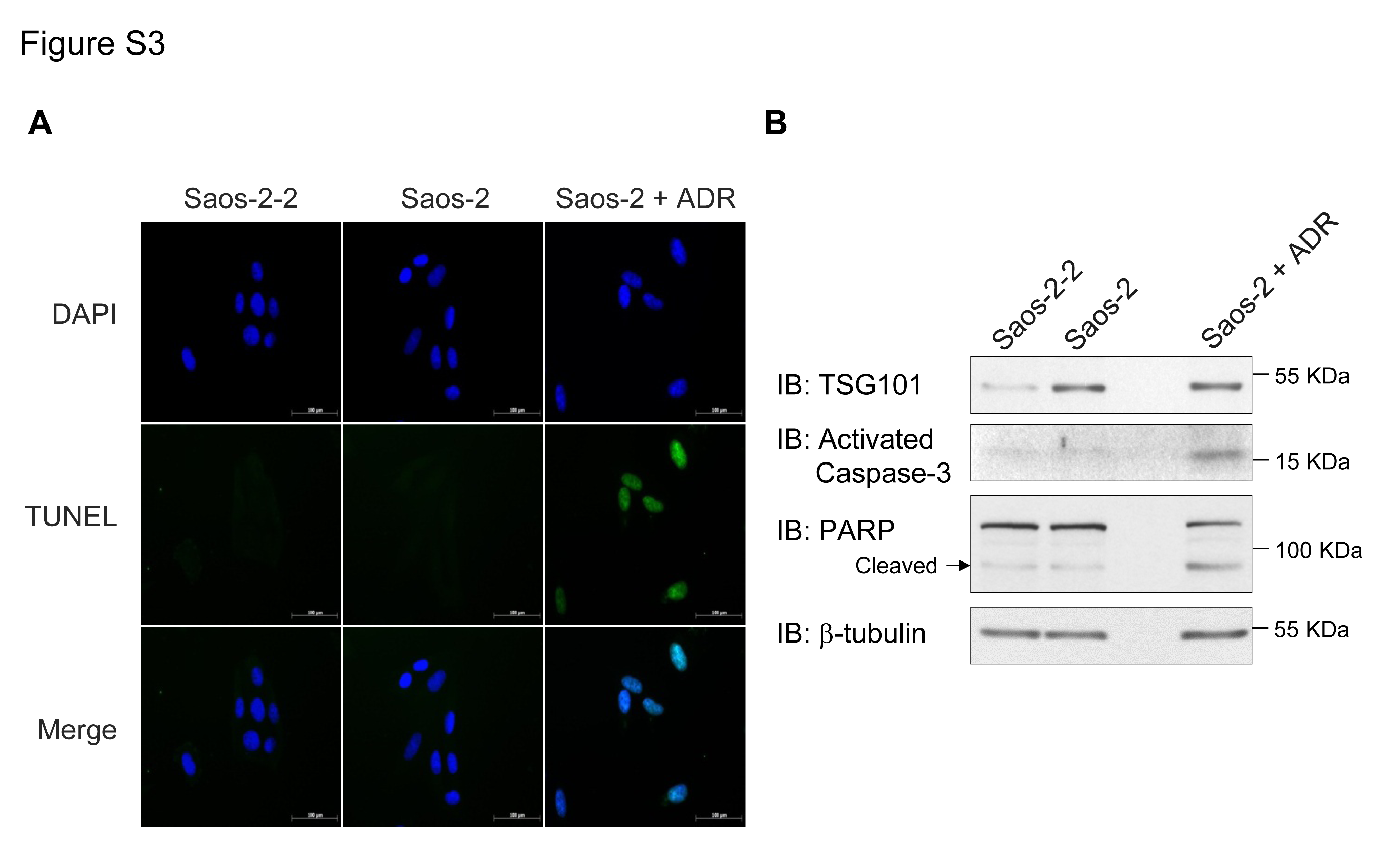

Supplement: Figure S3 — Saos-2-2 does not show a substantial increase of cell apoptosis compared to its parental cells. (A) Saos-2 and Saos-2-2 were analyzed for cell apoptosis by TUNEL assay. Saos-2 treated with Adriamycin (ADR, 1 µM), a DNA-damaging reagent, also were included as a positive control. Scale bars, 100 µm. (B) TSG101, Caspase-3, and Poly ADP-ribose polymerase (PARP) were examined in indicated cells by Western blot analyses. Activated Caspase-3 (17 KDa) and the cleavage form of PARP (85 KDa) both serve as index for the activation of apoptotic signal pathways. (TIF) [file pone.0079674.s003.tif]
