# Supplementary material for: Identification of TSG101 Functional Domains and p21 Loci Required for TSG101-Mediated p21 Gene Regulation
Source: PLoS One. 2013 Nov 11;8(11):e79674. doi: 10.1371/journal.pone.0079674 (PMC3823576; doi:10.1371/journal.pone.0079674)
Supplement: Table S1 — Oligonucleotide primers used in Chromatin Immunoprecipitation (ChIP) and quantitative RT-PCR (qPCR). (PDF) [file pone.0079674.s004.pdf]

Table S1.

ChIP	sequence
a- 5' primer	5'-GTTCAGGTGAGTGTAGGGTGTAG-3'
a- 3' primer	5'-TTTTTTTACTTGGAGAATG AGTTGGC-3'
b- 5' primer	5'-GGAGGTCAGGGGTGTGAGGTAGAT-3'
b- 3' primer	5'-GGAA GGAGGGAATTGGAGAGACTA-3'
c- 5' primer	5'-TGGTTTGGATGTATAGGAGCGAA GGTG-3'
c- 3' primer	5'-CTCCAAAATGACAAAAATGCCAAATAAC-3'
d- 5' primer	5'-CAG CCTGCTCCCTTGCCTTTTTCA-3'
d- 3' primer	5'-GACCCCACTCTAAGCCCCACTG C-3'
e- 5' primer	5'-GGGGGCTGGACTGGGCACTCTTGT-3'
e- 3' primer	5'-GCTCACCAC CACCACGACATTCAA-3'
f- 5' primer	5'-CTGAGATTGTGCCACTGCTGACTTT-3'
f- 3' primer	5'-CCAGAAAGCCAATCAGAGCCACAGCC-3'
**qPCR**	**sequence**
p21 qPCR-5' primer	5'-TCACTGTCTTGTACCCTTGTGC-3'
p21 qPCR-3' primer	5'-GGCGTTTGGAGTGGTAGAAA-3'
b-actin qPCR-5' primer	5'-GCACCCAGCACAATGAAGA-3'
b-actin qPCR-3' primer	5'-CGATCCACACGGAGTACTTG-3'
