# Supplementary material for: Identification of TSG101 Functional Domains and p21 Loci Required for TSG101-Mediated p21 Gene Regulation
Source: PLoS One. 2013 Nov 11;8(11):e79674. doi: 10.1371/journal.pone.0079674 (PMC3823576; doi:10.1371/journal.pone.0079674)
Supplement: Table S2 — List of genes that are statistically significant up-regulated or down-regulated in Saos-2-2 compared with its parental cells. Microarray data were deposited in GEO with an accession number GSE50808 (http://www.ncbi.nlm.nih.gov/geo/query/acc.cgi?acc=GSE50808), and analyzed by a software package “Limma” at http://bioconductor.org/packages/2.12/bioc/html/limma. html. Genes up-regulated or down-regulated more than 4-fold (with a p value less than 0.01) are shown. (PDF) [file pone.0079674.s005.pdf]

Table S2.

GenBank ID	Gene Symbol	Gene Name	Log2 Ratio	*P* value
Up-regulated				
AA115877	SERPINI1	Serpin peptidase inhibitor, clade I (neuroserpin), member 1	3.594	2.06E-04
AA683041	NPTX2	Neuronal pentraxin II	3.376	1.94E-05
AI732248	COL4A1	Collagen, type IV, alpha 1	3.361	2.61E-05
AA459895	CCDC64B	Coiled-coil domain containing 64B	3.170	1.39E-04
AA994913	GALNT12	UDP-N-acetyl-alpha-D-galactosamine:polypeptide N-acetylgalactosaminyltransferase 12 (GalNAc-T12)	3.105	4.28E-06
AA903339	RET	Ret proto-oncogene	3.036	2.75E-05
AA492170	NKX3-1	NK3 homeobox 1	2.957	1.00E-05
N50428	SLAIN1	SLAIN motif family, member 1	2.946	9.83E-06
AA457718	CLIC5	Chloride intracellular channel 5	2.887	1.02E-05
AA608524	MIB2	Mindbomb homolog 2 (Drosophila)	2.812	3.00E-05
AI732255	SFTPD	Surfactant protein D	2.796	1.48E-05
AI821374	SFTPA1	Surfactant protein A1	2.771	1.89E-05
AI276270	EMB	Embigin	2.742	1.14E-05
N22495	EMCN	Endomucin	2.730	1.10E-05
AW058501	COL4A4	Collagen, type IV, alpha 4	2.723	6.28E-04
R43270	PPP1R14C	protein phosphatase 1, regulatory (inhibitor) subunit 14C	2.679	1.16E-04

AI444994	JAK1	Janus kinase 1	2.643	3.36E-03
R51361	NFIB	Nuclear factor I/B	2.640	1.18E-05
AA169183	ST8SIA1	ST8 alpha-N-acetyl-neuraminide alpha-2,8-sialyltransferase 1	2.627	5.02E-04
AA425820	CCDC74A	Coiled-coil domain containing 74A	2.602	1.51E-05
AA228477	TARP	TCR gamma alternate reading frame protein	2.509	2.17E-05
AA977683	SGIP1	SH3-domain GRB2-like (endophilin) interacting protein 1	2.495	2.28E-05
T92782	SYK	Spleen tyrosine kinase	2.490	2.03E-05
H23524	SYT4	Synaptotagmin IV	2.479	4.37E-05
AI983123	BRDT	Bromodomain, testis-specific	2.467	4.02E-05
AI225111	GJB3	Gap junction protein, beta 3, 31kDa	2.453	2.13E-05
AA418392	KCNMB4	Potassium large conductance calcium-activated channel, subfamily M, beta member 4	2.452	7.58E-05
AA872098	NCF2	Neutrophil cytosolic factor 2	2.428	2.84E-05
AA603356	PLEKHH1	Pleckstrin homology domain containing, family H (with MyTH4 domain) member 1	2.427	3.28E-05
AA863023	SCGB1C1	Secretoglobin, family 1C, member 1	2.416	3.85E-05
AA464180	BEX2	Brain expressed X-linked 2	2.413	2.01E-05
AI732254	GKN2	Gastrokine 2	2.413	2.54E-03
AI732256	AGR3	Anterior gradient homolog 3 (Xenopus laevis)	2.407	3.59E-05
R54212	XKR4	XK, Kell blood group complex subunit-related family, member 4	2.396	8.57E-05
AA420679	XCL2	Chemokine (C motif) ligand 2	2.358	3.20E-05
AA169475	SNAPC5	Small nuclear RNA activating complex, polypeptide 5, 19kDa	2.353	5.94E-05
AI740542	FXYD1	FXYD domain containing ion transport regulator 1	2.351	5.59E-03
N23941	CDKN1A	Cyclin-dependent kinase inhibitor 1A (p21, Cip1)	2.343	3.35E-04
AI193221	ZNF720	Zinc finger protein 720	2.342	4.04E-05

AA443593	ACRBP	Acrosin binding protein	2.336	5.84E-05
AI732298	DNAJA4	DnaJ (Hsp40) homolog, subfamily A, member 4	2.333	3.55E-05
AI732290	MYOT	Myotilin	2.322	6.92E-04
AA479055	PUS1	Pseudouridylate synthase 1	2.322	6.92E-04
W23758	USP54	Ubiquitin specific peptidase 54	2.316	7.11E-05
AA490240	KRTAP19-1	Keratin associated protein 19-1	2.299	5.96E-05
N45301	CALCRL	Calcitonin receptor-like	2.299	3.47E-05
H99722	ANO5	Anoctamin 5	2.293	4.59E-05
AA629873	UBA6	Ubiquitin-like modifier activating enzyme 6	2.289	8.66E-05
N69335	COL9A1	Collagen, type IX, alpha 1	2.286	2.81E-05
R70505	P2RY2	Purinergic receptor P2Y, G-protein coupled, 2	2.267	3.63E-05
AI732322	CKAP2	Cytoskeleton associated protein 2	2.230	4.95E-05
AA977633	NRK	Nik related kinase	2.225	7.99E-05
W60582	BEX1	Brain expressed, X-linked 1	2.222	2.78E-03
AA460698	PAMR1	Peptidase domain containing associated with muscle regeneration 1	2.213	5.64E-05
AA412443	LCN10	Lipocalin 10	2.212	5.15E-05
AA425730	ILDR1	Immunoglobulin-like domain containing receptor 1	2.193	3.75E-05
AA988559	SCGB3A2	Secretoglobin, family 3A, member 2	2.192	9.59E-05
AA410381	SNCAIP	Synuclein, alpha interacting protein	2.189	3.65E-04
H15099	ANK2	Ankyrin 2, neuronal	2.178	3.81E-05
AI821350	SFTPA2B	surfactant protein A2B	2.177	6.84E-05
AA534438	SPINK4	Serine peptidase inhibitor, Kazal type 4	2.173	9.24E-05
AA745686	BHMT2	Betaine--homocysteine S-methyltransferase 2	2.171	3.42E-04

AA172188	PCDHA6	Protocadherin alpha 6	2.171	3.89E-05
AA029949	NPNT	Nephronectin	2.170	4.81E-05
N21659	MORN4	MORN repeat containing 4	2.166	1.21E-04
AA429034	PIK3IP1	Phosphoinositide-3-kinase interacting protein 1	2.157	2.01E-04
AA100297	LGALS4	Lectin, galactoside-binding, soluble, 4	2.148	1.20E-04
AA746038	GPR110	G protein-coupled receptor 110	2.145	1.19E-04
AA996091	FSIP1	Fibrous sheath interacting protein 1	2.136	9.36E-05
AA228476	SLC26A7	Solute carrier family 26, member 7	2.130	1.95E-04
AA131664	PRELP	Proline/arginine-rich end leucine-rich repeat protein	2.130	1.44E-03
AA101678	MYCN	V-myc myelocytomatosis viral related oncogene, neuroblastoma derived (avian)locus	2.127	4.78E-05
AA488171	DAB2IP	DAB2 interacting protein	2.126	6.51E-03
AA233805	DAB1	Disabled homolog 1 (Drosophila)	2.121	9.89E-04
AA632429	LRRFIP1	Leucine rich repeat (in FLII) interacting protein 1	2.119	7.48E-05
AA996097	PANX2	Pannexin 2	2.116	2.10E-04
AA167273	SDC3	Syndecan 3	2.100	6.08E-05
AA525000	WDFY3	WD repeat and FYVE domain containing 3	2.099	6.38E-05
AA564296	PIP	Prolactin-induced protein	2.085	2.06E-04
AA149051	PGR	Progesterone receptor	2.080	5.36E-05
R42056	LGI2	Leucine-rich repeat LGI family, member 2	2.073	2.59E-04
AA514424	FAM3D	Family with sequence similarity 3, member D	2.065	9.00E-05
H85454	KCNS1	Potassium voltage-gated channel, delayed-rectifier, subfamily S, member 1	2.064	9.77E-05
AA457474	RAMP2	Receptor (G protein-coupled) activity modifying protein 2	2.059	1.47E-04
R02333	UBXN10	UBX domain protein 10	2.058	4.10E-04

AA451911	GPR126	G protein-coupled receptor 126	2.054	1.18E-04
AI769855	DEFB1	Defensin, beta 1	2.048	1.91E-04
AA180237	TCF7L1	Transcription factor 7-like 1 (T-cell specific, HMG-box)	2.038	7.53E-05
AA973944	PGBD5	PiggyBac transposable element derived 5	2.037	7.26E-04
AI732898	CLEC5A	C-type lectin domain family 5, member A	2.036	7.78E-05
AA907365	TMEM146	Transmembrane protein 146	2.034	1.29E-04
AA875890	GRIN2A	Glutamate receptor, ionotropic, N-methyl D-aspartate 2A	2.026	7.24E-05
AI273601	KPRP	Keratinocyte proline-rich protein	2.025	6.06E-05
AA621001	LRRTM3	Leucine rich repeat transmembrane neuronal 3	2.020	7.88E-05
AI732312	EPHB1	EPH receptor B1	2.019	1.53E-04
R54590	PLEKHA6	Pleckstrin homology domain containing, family A member 6	2.016	3.43E-04
AA469952	ALLC	Allantoicase	2.014	4.56E-04
AI016902	AICDA	Activation-induced cytidine deaminase	2.011	1.14E-04
AA928139	PCDH17	Protocadherin 17	2.008	1.32E-04
R99004	DHRS7	Dehydrogenase/reductase (SDR family) member 7	2.000	4.99E-04
AA458634	NQO1	NAD(P)H dehydrogenase, quinone 1	2	1.45E-03

Down-regulated

T72089	NNMT	Nicotinamide N-methyltransferase	-4.602	8.92E-06
AA449321	LRRC15	Leucine rich repeat containing 15	-4.387	5.98E-07
AA457688	IMPA1	Inositol(myo)-1(or 4)-monophosphatase 1	-4.303	2.74E-05
R42630	PCSK1	Proprotein convertase subtilisin/kexin type 1	-3.459	8.79E-05
AA055835	CAV1	Caveolin 1, caveolae protein, 22kDa	-3.251	5.88E-06
T95052	CASP1	Caspase 1, apoptosis-related cysteine peptidase (interleukin 1, beta, convertase)	-3.168	1.93E-04
H59861	THBD	Thrombomodulin	-3.080	5.03E-05
N62462	CFI	Complement factor I	-3.027	6.99E-06
AA449715	SRPX	Sushi-repeat containing protein, X-linked	-3.018	7.44E-06
AA176812	POLR1E	Polymerase (RNA) I polypeptide E, 53kDa	-3.000	1.86E-04
H82419	PTPRA	Protein tyrosine phosphatase, receptor type, A	-2.907	2.19E-04
AA425746	RUNX1	Runt-related transcription factor 1	-2.867	7.92E-05
AI346653	THY1	Thy-1 cell surface antigen	-2.819	1.54E-04
H08561	IGFBP5	Insulin-like growth factor binding protein 5	-2.812	1.28E-05
AI148233	IL11	Interleukin 11	-2.777	1.07E-05
AA447684	SPRR1B	Small proline-rich protein 1B	-2.755	2.87E-04
AA633901	TGFBI	Transforming growth factor, beta-induced, 68kDa	-2.739	1.31E-05
AA905329	SENP1	SUMO1/sentrin specific peptidase 1	-2.721	1.49E-05
R12373	PON1	Paraoxonase 1	-2.705	1.43E-05
H90764	SERPINA6	Serpin peptidase inhibitor, clade A (alpha-1 antiproteinase, antitrypsin), member 6	-2.667	2.90E-04
AA098867	NRP1	Neuropilin 1	-2.661	1.10E-05
AA173525	ZNF654	zinc finger protein 654	-2.630	1.20E-05

H91456	NR1H4	Nuclear receptor subfamily 1, group H, member 4	-2.600	3.37E-04
H20814	ARL3	ADP-ribosylation factor-like 3	-2.585	4.02E-04
AA464176	PIK3CG	Phosphoinositide-3-kinase, catalytic, gamma polypeptide	-2.544	1.47E-05
AI191504	AK7	Adenylate kinase 7	-2.500	9.42E-05
R56092	MYT1L	Myelin transcription factor 1-like	-2.500	6.07E-03
R73909	PSG11	Pregnancy specific beta-1-glycoprotein 6	-2.472	3.90E-03
AA088420	PPARG	Peroxisome proliferator-activated receptor gamma	-2.468	1.99E-05
N71920	ODZ2	odz, odd Oz/ten-m homolog 2 (Drosophila)	-2.461	6.05E-05
AA131406	CXCL9	Chemokine (C-X-C motif) ligand 9	-2.459	5.18E-04
N34362	RGS5	Regulator of G-protein signaling 5	-2.453	3.24E-04
AA485373	VMP1	Vacuole membrane protein 1	-2.405	8.13E-05
N22620	AMIGO2	Adhesion molecule with Ig-like domain 2	-2.360	4.93E-05
R56082	SV2B	Synaptic vesicle glycoprotein 2B	-2.324	3.17E-05
H65734	KLF1	Kruppel-like factor 1 (erythroid)	-2.322	6.92E-04
AA669451	MKRN1	Makorin ring finger protein 1	-2.322	6.92E-04
AA416767	CCDC80	Coiled-coil domain containing 80	-2.291	4.90E-05
W48852	GREM1	Gremlin 1	-2.258	3.71E-03
AA454652	F2RL1	Coagulation factor II (thrombin) receptor-like 1	-2.252	1.82E-04
AA972352	PDLIM3	PDZ and LIM domain 3	-2.238	4.24E-05
AA029997	COL4A5	Collagen, type IV, alpha 5	-2.230	4.64E-03
N67039	CDK6	Cyclin-dependent kinase 6	-2.217	3.54E-05
AA995128	FIGF	C-fos induced growth factor (vascular endothelial growth factor D)	-2.216	1.59E-04
R39044	RAB27B	RAB27B, member RAS oncogene family	-2.214	3.60E-05

AA779870	FHAD1	Forkhead-associated (FHA) phosphopeptide binding domain 1	-2.152	1.82E-03
R41754	TMEM132B	Transmembrane protein 132B	-2.122	1.91E-04
R69567	PSG5	pregnancy specific beta-1-glycoprotein 5	-2.113	8.34E-04
AI828190	PLAUR	Plasminogen activator, urokinase receptor	-2.094	7.80E-04
T87622	SAP30	Sin3A-associated protein, 30kDa	-2.085	6.60E-03
H84369	POPDC3	Popeye domain containing 3	-2.069	1.66E-03
AA703531	IL24	Interleukin 24	-2.058	6.89E-05
AA056415	FBN1	Fibrillin 1	-2.051	1.72E-04
AA010664	TAGLN	Transgelin	-2.048	5.66E-05
AA455235	ALDH1A3	Aldehyde dehydrogenase 1 family, member A3	-2.032	4.82E-03
N79051	ROS1	C-ros oncogene 1 , receptor tyrosine kinase	-2.020	1.57E-04
H22854	REPS2	RALBP1 associated Eps domain containing 2	-2.015	1.42E-03
AA702808	ADAM12	ADAM metallopeptidase domain 12	-2.014	2.80E-04
H23197	MOBP	Myelin-associated oligodendrocyte basic protein	-2.012	6.06E-03
AA777187	CYR61	Cysteine-rich, angiogenic inducer, 61	-2.011	6.74E-04
H17080	MBP	Myelin basic protein	-2	1.45E-03
H37761	NR4A3	Nuclear receptor subfamily 4, group A, member 3	-2	1.45E-03
